# Bioinformatics Analysis and Experimental Findings Reveal the Therapeutic Actions and Targets of *Cyathulae Radix* Against Type 2 Diabetes Mellitus

**DOI:** 10.1155/2024/5521114

**Published:** 2024-11-05

**Authors:** Xi Zhang, Zijin Sun, Wenlong Sun, Yueming Li, Fei Gao, Fei Teng, Zhenxu Han, Yanting Lu, Shuo Zhang, Lingru Li

**Affiliations:** ^1^College of Traditional Chinese Medicine, Beijing University of Chinese Medicine, Beijing 102488, China; ^2^School of Life Sciences and Medicine, Shandong University of Technology, Zibo 255049, China; ^3^College of Traditional Chinese Medicine, Shandong University of Traditional Chinese Medicine, Jinan 250014, China; ^4^National Institute of TCM Constitution and Preventive Medicine, Beijing University of Chinese Medicine, Beijing 100029, China

**Keywords:** bioinformatics, *Cyathulae Radix*, molecular mechanisms, therapeutic targets, traditional Chinese medicine, type 2 diabetes mellitus

## Abstract

**Objective:** This study elucidated the mechanistic role of *Cyathulae Radix* (CR) in type 2 diabetes mellitus (T2DM) through bioinformatics analysis and experimental validation.

**Methods:** Components and targets of CR were retrieved from the traditional Chinese medical systems pharmacology, while potential T2DM targets were obtained from GeneCards and Online Mendelian Inheritance in Man databases. Intersecting these datasets yielded target genes between CR and T2DM. Differential genes were used for constructing a protein–protein interaction network, followed by Gene Ontology (GO) and Kyoto Encyclopedia of Genes and Genomes (KEGG) enrichment analyses. Molecular docking and dynamics simulations were performed using AutoDock and GROMACS, respectively, and in vitro experiments validated the results. Experiments evaluated the effect of CR on T2DM pancreatic *β*-cells.

**Results:** Bioinformatics analysis identified four active compounds of CR, 157 related genes, and 5431 T2DM target genes, with 141 shared targets. Key targets such as JUN, MAPK1, and MAPK14 were identified through topological analysis of the PPI network. GO analysis presented 2663 entries, while KEGG analysis identified 161 pathways. The molecular docking results demonstrated favorable binding energy between the core components and the core proteins. Among them, JUN-rubrosterone, MAPK1-rubrosterone, and MAPK14-rubrosterone deserved further investigation. Molecular dynamics results indicated that all of them can form stable binding interactions. CR could inhibit the expression of JUN, MAPK1, and MAPK14, promote insulin secretion, alleviate apoptosis, and regulate autophagy in INS-1 cells.

**Conclusion:** This study suggests CR approach to T2DM management by multitarget and multipathway provides a scientific basis for further research on the hypoglycemic effect of CR.

## 1. Introduction

Type 2 diabetes mellitus (T2DM) is a chronic systemic metabolic disorder characterized by elevated blood glucose levels caused by insulin resistance (IR) and/or impaired insulin secretion [1]. According to the International Diabetes Federation (IDF), the number of adults with diabetes worldwide reached 537 million in 2021 [2]. The increasing prevalence and mortality rates of diabetes have imposed significant public health and economic burdens. Currently, there is no cure for T2DM. Treatment approaches mainly involve using antidiabetic drugs and insulin to maintain blood glucose levels as close to regular as possible and to delay or prevent the development of diabetes-related complications [3]. Although these drugs have shown efficacy, their mechanisms of action primarily target individual compounds, and adverse reactions have been reported. Traditional Chinese medicine, as complementary and alternative medicine, has a long history of meaningful efficacy in treating T2DM, providing a new option for T2DM management. Research and development of new drugs based on traditional Chinese medicine is essential in T2DM research.

*Cyathulae Radix* (CR), also known as “Chuan Niu Xi,” refers to the dried roots of *Cyathula officinalis* Kuan. It is a well-known Chinese herbal medicine mentioned in the “Shen Nong Ben Cao Jing” (Shennong Materia Medica). CR was included in the Pharmacopoeia of the People's Republic of China (Pharmacopoeia Commission China, 2020). According to traditional Chinese medicine theory, CR promotes blood circulation, removes blood stasis, promotes joint mobility, and promotes diuresis. CR is an ingredient in the classic traditional Chinese medicine formula “Yu Nu Compound,” commonly used for treating diabetes, with a history of thousands of years in China [4]. CR was widely used in clinical practice for treating various infectious diseases and possesses anti-inflammatory properties [5]. Several natural compounds in CR, such as triterpenoid saponins, steroid ketones, polysaccharides, and other compounds, exhibit various pharmacological activities in vivo and in vitro, including regulating blood viscosity, improving microcirculation, and enhancing immune function. The application of blood-activating herbs like CR might reduce the occurrence of diabetes [5, 6]. CR demonstrates a beneficial effect in lowering blood glucose and improving symptoms in treating T2DM. However, due to the multiple components and multitarget properties of traditional Chinese herbal medicine, the mechanisms of its active compounds on T2DM have not been fully elucidated.

Bioinformatics is a multidisciplinary field that integrates network pharmacology, molecular docking, and molecular dynamics. Network pharmacology enables the identification of crucial target molecules for drug treatment of diseases at multiple levels. It is considered an effective research method for exploring the underlying molecular mechanisms of diseases. The therapeutic benefits of herbal medicine are attributed to the combined or synergistic effects of various components and targets [7]. Molecular docking is a technique that combines drugs with protein receptors and assesses their affinity based on binding capacity [8]. Molecular dynamics, based on classical mechanics, quantum mechanics, and statistical mechanics, utilizes computer numerical methods to solve molecular system motion equations and simulate the structure and properties of molecular systems. Therefore, the characteristics of herbal medicine align well with bioinformatics, consistent with the holistic perspective of traditional Chinese medicine in treating diseases [9, 10]. This makes it a rational predictive method for investigating the mechanisms of herbal medicine. This study is aimed at examining the therapeutic effects of CR in treating T2DM by investigating the main bioactive components, molecular targets, and pathways involved. This was achieved through bioinformatics analysis and experimental validation to obtain valuable insights into the mechanisms underlying the hypoglycemic effects of CR.

## 2. Materials and Methods

### 2.1. Collection of Bioactive Substances From CR and Prediction of Relevant Targets

The drug components of CR and their corresponding targets were obtained from the Traditional Chinese Medicine Systems Pharmacology Database and Analysis Platform (TCMSP) (https://old.tcmsp-e.com/tcmsp.php) [11]. Many compounds in traditional Chinese medicine are unsuitable for reaching specific protein targets within cells due to their pharmacological characteristics. Therefore, the selection of appropriate screening indicators is crucial in bioinformatics analysis. One of the most important pharmacokinetic parameters of a drug is the oral bioavailability (OB) value, which is defined as the ratio of the quantity of active ingredients absorbed into the circulatory system at the site of action to the total quantity of active ingredients. Compounds with an OB ≥ 30% were well absorbed and metabolized slowly [12]. Additionally, the drug-likeness (DL) value indicates whether a molecule contains specific functional groups or exhibits physicochemical properties similar to most drugs. Compounds with DL ≥ 0.18 were chemically suitable for drug development. In this study, compounds meeting the thresholds of OB ≥ 30% and DL ≥ 0.18 were selected, and their corresponding protein targets were included in the study. After screening the active ingredients, identifying the targets for each component is crucial. We used the UniProtKB search function in the UniProt database (https://uniprot.org/) and restricted the input to “human species” to obtain the official gene names for each protein [13, 14].

### 2.2. Discovery and Prediction of Targets Associated With T2DM

Using “type 2 diabetes mellitus” as the search keyword in the GeneCards (https://www.genecards.org/) and Online Mendelian Inheritance in Man (OMIM) databases (https://www.omim.org/), disease-related targets for T2DM were identified. A higher score in the GeneCards database indicates a stronger correlation between the target and the disease. Hence, targets with a relevance score ≥ 10 were identified as T2DM targets. After merging the target lists from both databases and removing duplicates, the resulting targets were consolidated as the candidate disease targets.

### 2.3. Analysis of Common Targets Between CR and T2DM

The targets of CR collected intersected with the targets of T2DM. Using R 4.0.2 and the relevant R packages, a Venn diagram was generated to visualize the intersection. The intersecting targets were considered as potential intervention targets of CR for T2DM.

### 2.4. Protein–Protein Interaction (PPI) Data Analysis and Identification of Core Targets

To further identify key regulatory targets, the intersecting targets obtained previously were submitted to the STRING database (https://stringdb.org/), known for its comprehensive protein and biological data. The analysis focused on “Homo sapiens” as the species, with a minimum interaction threshold of 0.9. Isolated nodes were concealed. The CytoNCA plugin was employed to calculate the betweenness centrality, closeness centrality, degree centrality (DC), edge clustering coefficient, local average connectivity, and network centrality values for each node. Topological analysis was performed twice to derive the core network of the PPI network.

### 2.5. Gene Ontology (GO) Functional Analysis and Kyoto Encyclopedia of Genes and Genomes (KEGG) Pathway Enrichment Analysis

The collected common targets were converted to their corresponding Entrez IDs for GO functional analysis and KEGG pathway analysis. Enrichment analysis was performed by R 4.0.2 software and relevant R packages (clusterProfiler, http://org.Hs.eg.db, enrichplot, ggplot2, pathview, ggnewscale, and stringr), and the most significant GO terms and KEGG signaling pathways were chosen based on a screening threshold of *p* < 0.05. The results were visualized by generating bar graphs to analyze the possible mechanisms of T2DM treatment with CR from the perspective of biological functions and signaling pathways.

### 2.6. Molecular Docking

To obtain reliable receptor–ligand complexes, we employed molecular docking for our study. Molecular docking is a commonly used method in drug discovery. It accurately predicts suitable binding sites and conformations of small molecule ligands and evaluates binding energies. The docking process was performed using AutoDock Tools 1.5.6 and AutoDock Vina. These were widely used molecular docking strategies that have been validated to enhance the speed and accuracy of molecular docking. The docking process involved the following steps: Firstly, three-dimensional structures of proteins were retrieved from the Protein Data Bank (PDB) (http://www.rcsb.org/pdb) and converted into PDBQT files. Then, small molecule ligand files were downloaded from the PubChem database (http://pubchem.ncbi.nlm.nih.gov/), and modifications were made to the ligands, which were saved as PDBQT files. The receptor–ligand complexes were subjected to docking after setting parameters such as binding pockets and docking times.

### 2.7. Molecular Dynamics Simulations and Evaluation

To ensure the stability of the obtained receptor–ligand complexes, molecular dynamics simulations were performed on the top three receptor–ligand complexes ranked by binding energy using GROMACS 2021.2. The small molecule (ligand) was processed using SwissParam. The protein topology file was generated using the AMBER99SB-ILDN force field, while the ligand topology file was generated using the AMBER force field through the ACPYPE script. Molecular dynamics simulations were conducted in a triclinic box filled with TIP3 water molecules, applying periodic boundary conditions. Sodium and chloride ions were added to the system to neutralize the charge. To minimize collisions between the protein and small molecule, the system was optimized using the steepest descent method to reach the state of optimal potential energy. Before the molecular dynamics simulations, the complexes were minimized for 1000 steps and equilibrated by running NVT and NPT simulations for 100 ps. Subsequently, each system underwent 100 ns molecular dynamics simulations at a temperature of 310 K and a pressure of 1.0 bar under periodic boundary conditions. Evaluation and calculations were performed for each system.

### 2.8. Calculation of Molecular Mechanics Poisson–Boltzmann Surface Area (MM-PBSA) Combined Free Energy

To complement the results obtained from molecular dynamics trajectories, the affinity between the protein and small molecule ligand was quantitatively assessed by calculating binding free energy. MM-PBSA is widely employed to calculate binding free energies and predict the stability of receptor–ligand complexes after molecular dynamics simulations [15]. The binding free energy between the protein and small molecule ligand for all systems was computed using the MM-PBSA method. The calculation was performed with a 100 ns molecular dynamics trajectory according to the following formula:

$$\begin{array}{cc} \; & \Delta G_{\text{bind}}=\Delta G_{\text{complex}}-\left(\Delta G_{\text{receptor}}+\Delta G_{\text{ligand}}\right)=\Delta H-\text{T}\Delta \text{S}\\ \Delta H=\Delta E_{\text{MM}}+\Delta G_{\text{sol}}\\ \Delta E_{\text{MM}}=\Delta E_{\text{ele}}+\Delta E_{\text{vdW}}+\Delta E_{\mathrm{int}} \end{array}$$

Finally, merge the transformation formula to get

 $$\begin{array}{c} \Delta {\text{G}}_{\text{bind}}=\Delta {\text{E}}_{\mathrm{int}}+\Delta {\text{E}}_{\text{vdW}}+\Delta {\text{E}}_{\text{ele}}+\Delta {\text{G}}_{\text{PB}}+\Delta {\text{G}}_{\text{SA}} \end{array}$$

Δ*G*_bind_ represented the binding free energy, Δ*G*_complex_ was the binding free energy of the complex, Δ*G*_receptor_ was the receptor binding free energy, Δ*G*_ligand_ was the ligand binding free energy, *T*Δ*S* was the system entropy change, Δ*H* was the enthalpy, Δ*E*_MM_ was the gas-phase energy, Δ*G*_sol_ was the solvation energy, Δ*G*_PB_ was the polar solvation contribution, and Δ*G*_SA_ was the nonpolar solvation free energy. In the above formula, Δ*E*_MM_ was decomposed into Δ*E*_ele_, Δ*E*_vdW_, and Δ*E*_int_ (representing electrostatic energy, van der Waals, and internal energy, respectively). Δ*E*_int_ comprised bond energy, angle energy, and torsional energy. In this study, the conformational structures of the protein and small molecule ligand were obtained from a single molecular dynamics simulation trajectory (complex trajectory only), considering the protein-small molecule ligand structure as a rigid body. This means that Δ*E*_int_ between the complex and individual molecules can mutually cancel out since this energy term was calculated from the same molecular dynamics simulation trajectory. Therefore, this study focused on Δ*E*_ele_ and Δ*E*_vdW_.

### 2.9. Cell Culture

Cell culture experiments were performed according to the guidelines and regulations of Shandong University of Traditional Chinese Medicine. INS-1 cells were obtained from the cell bank of Cyagen (Guangdong, China). The normal cell culture medium was prepared using low-glucose Dulbecco's Modified Eagle Medium (DMEM) supplemented with 5.5 mmol/L glucose (HyClone, USA) and 10% fetal bovine serum (Lonsera, China) [16]. The cells were maintained at 37°C in a humidified atmosphere containing 5% carbon dioxide. The model group was cultured in a medium containing 30 mmol/L glucose [17]. CR was purchased from the Traditional Chinese Medicine Pharmacy at the Affiliated Hospital of Shandong University of Traditional Chinese Medicine (Jinan, China). It was decocted twice with boiling deionized water at a ratio of 30:1 and then 10:1 (w/v) for 30 min each time. The solution was filtered through 0.22 *μ*m filters and stored at −20°C [18]. The experimental groups included the control group (5.5 mmol/L glucose), the high-glucose model group (30 mmol/L glucose), the low-dose CR group (30 mmol/L glucose and 5 *μ*g/mL CR), the medium-dose CR group (30 mmol/L glucose and 10 *μ*g/mL CR), and the high-dose CR group (30 mmol/L glucose and 20 *μ*g/mL CR).

### 2.10. CCK-8 Assay

Cell viability was determined using the CCK-8 kit (UUBIOTM, China). INS-1 cells were seeded at a density of 1 × 10^4^ cells per well in every 96-well plate. After 12 h of incubation, the cells were treated with different concentrations for 24, 48, and 72 h. Subsequently, 10 *μ*L of CCK-8 was added to each well, and the plate was incubated for 1–4 h. Finally, absorbance was measured at a wavelength of 450 nm. The experiment was repeated 3 times.

### 2.11. Insulin Assay

Cells were seeded at a density of 1 × 10^4^ cells per well in every 96-well plate. The intervention groups were used: the control, model, and CR group. Each group was intervened for 48 h and repeated 3 times. Insulin levels were measured using an enzyme-linked immunosorbent assay (ELISA) kit (Elabscience Biotechnology Co., Ltd., China).

### 2.12. Transmission Electron Microscopy (TEM) Observation

The samples were prefixed with 3% glutaraldehyde and then fixed with 1% osmium tetroxide. Subsequently, the samples were dehydrated using a graded series of acetone and embedded in Ep812 resin, and semithin sections were stained with toluidine blue for optical localization. Ultrathin sections were cut with a diamond knife and stained with uranyl acetate and lead citrate for observation using the JEM-1400FLASH transmission electron microscope.

### 2.13. RNA Extraction and Quantitative Polymerase Chain Reactions (qPCR)

Total RNA was extracted from INS-1 cells using an RNA extraction kit (Shandong Sparkjade, China), and the RNA purity was determined using the Nanodrop 2000c spectrophotometer (Thermo Fisher Scientific, United States). The total RNA was reverse transcribed into cDNA using a reverse transcription kit (Shandong Sparkjade, China). qPCR was performed using the SYBR Green I reagent kit (Shandong Sparkjade, China) on the LC480 system (Roche, Germany) for 40 cycles (94°C for 20 s, 60°C for 20 s, and 72°C for 30 s). All steps, parameter settings, and instrument selections were based on the manufacturer's proposal [19]. Glyceraldehyde-3-phosphate dehydrogenase (GAPDH) was used as an internal reference gene, and the 2^−ΔΔCT^ method was employed. The specific primer sequences are listed in Table 1.

### 2.14. Statistical Analysis

Statistical analysis was performed using SPSS 26.0 software, and graphs were generated using GraphPad Prism 9.0. Group differences were determined using unpaired *t*-tests or one-way analysis of variance (ANOVA). Differences were considered significant at *p* < 0.05.

## 3. Results

### 3.1. Collection of Active Compounds for CR

Active compounds of CR were retrieved from the TCMSP, resulting in 41 related compounds. Using screening thresholds of OB ≥ 30% and DL ≥ 0.18, duplicate entries were removed, resulting in a final selection of four components. They were beta vulgarin, quercetin, beta-sitosterol, and rubrosterone. These components were considered as the effective substances of CR. The details of each component can be found in Table S1.

### 3.2. Prediction of Target Proteins for CR

Prediction of target proteins for the bioactive components of CR was performed using the TCMSP. After merging and eliminating duplicates, 157 related genes were obtained. These genes represent the target proteins of the bioactive components of CR in the human body.

### 3.3. Prediction of Target Genes for T2DM

Searches were conducted in the GeneCards and OMIM databases, resulting in 5019 target genes from the GeneCards database and 519 target genes from the OMIM database. After taking the union of these obtained targets, 5431 target genes related to T2DM were identified.

### 3.4. Analysis of Shared Targets Between CR and T2DM

**Figure 1:**
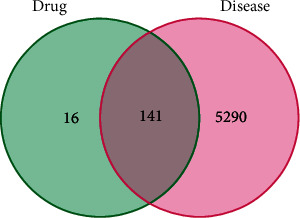
Venn diagram of the intersecting targets in CR and T2DM.

After intersecting the target genes of CR with those of T2DM, we obtained 141 shared targets, which were the effective sites of CR in treating T2DM (refer to Table S2). The Venn diagram illustrating the disease and drug targets is shown in Figure 1.

### 3.5. PPI Network Construction and Core Target Selection

**Figure 2:**
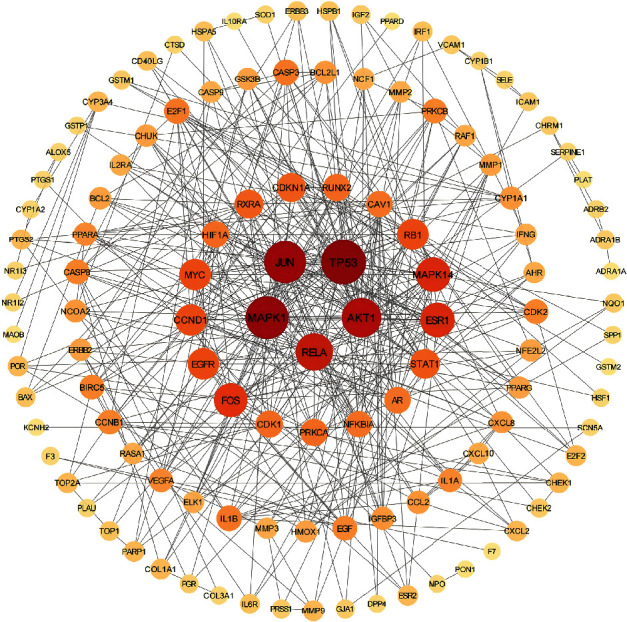
PPI network.

PPI analysis was performed to explore the interactions between the target genes at the protein level. After uploading the data to the STRING database, we obtained a PPI network consisting of 118 nodes and 440 edges, with an average node degree of 6.24 (Figure S1). Subsequently, we downloaded the TSV file of the PPI network and visualized it using Cytoscape 3.9.0 software (Figure 2). Based on topological analysis, we considered genes with these parameters exceeding the median as having important roles in the network. We performed this operation twice to identify the core network of the PPI network. Ultimately, we selected the Top 3 genes based on DC, namely Jun, MAPK1, and MAPK14, as the core targets.

### 3.6. GO and KEGG Enrichment Analysis

**Figure 3:**
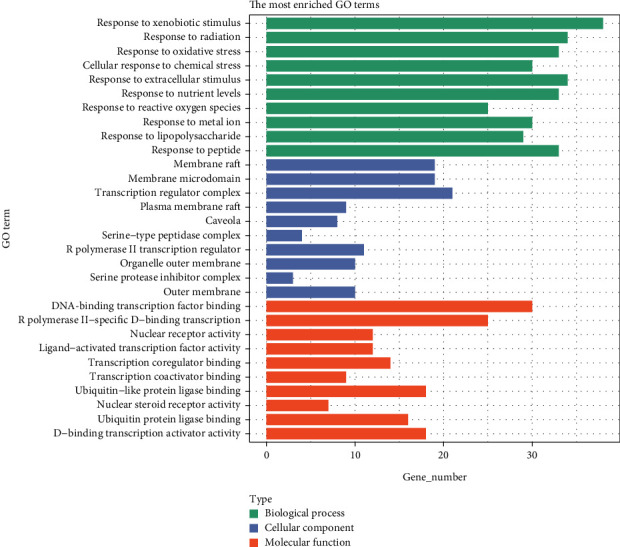
GO enrichment analysis of target genes.

To elucidate the potential mechanisms and biological pathways involved in CR treating T2DM, we conducted GO enrichment analysis and KEGG pathway enrichment analysis for the 141 target genes. The GO analysis included biological processes (BP), cellular components (CC), and molecular functions (MF). In this study, we obtained 2663 entries (*p* < 0.05), including 2333 BP entries, 83 CC entries, and 247 MF entries. The bar chart in Figure 3 displays the Top 30 enriched terms in the categories of BP, CC, and MF. The results indicate that the main pathways targeted by CR in treating T2DM were related to BP, such as response to xenobiotic stimulus (GO:0009410), response to radiation (GO:0009314), response to oxidative stress (GO:0006979), cellular response to chemical stress (GO:0062197), and response to extracellular stimulus (GO:0009991). In terms of CC, the enriched terms include membrane raft (GO:0044860), membrane microdomain (GO:0098857), transcription regulator complex (GO:0005667), plasma membrane raft (GO:0044853), and caveola (GO:0005901). The MF category includes terms such as DNA–binding transcription factor binding (GO:0140297), RNA polymerase II-specific DNA–binding transcription factor binding (GO:0061629), nuclear receptor activity (GO:0004879), ligand-activated transcription factor activity (GO:0098531), and transcription coregulator binding (GO:0001221).

**Figure 4:**
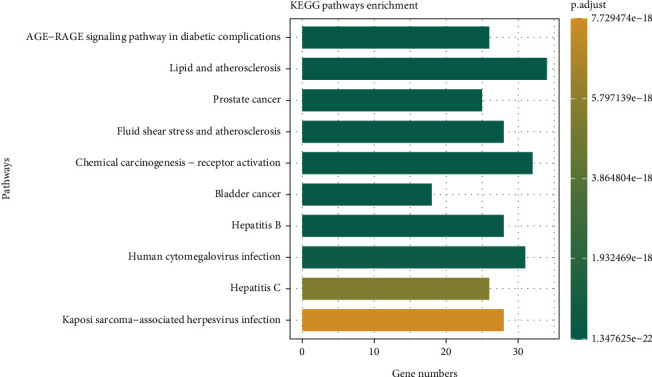
KEGG enrichment analysis of signaling pathways.

KEGG pathway analysis was performed to study functions and signal transduction pathways in CR treating T2DM. One hundred sixty-one statistically significant pathways (*p* < 0.05) were identified, with the Top 3 pathways being the AGE-RAGE signaling pathway in diabetic complications (hsa04933), lipid and atherosclerosis (hsa05417), and prostate cancer (hsa05215). The top 10 enriched pathways were visualized, and the results are presented in the form of a bar chart in Figure 4. Longer bars represented more genes enriched in the pathway. The color indicated the significance level, with a greener color indicating a smaller *p* value. Therefore, it could be concluded that CR exerts essential effects in treating T2DM through multiple targets and pathways.

### 3.7. Molecular Docking Results

Molecular docking analysis was performed to investigate the binding of the Top 3 targets (JUN, MAPK1, and MAPK14) with all 4 active compounds. The docking was conducted for each target separately, resulting in 12 combinations and the successful docking of 12 receptor–ligand pairs. The binding energies were frequently calculated to evaluate the degree of interaction between the components and protein targets. It is generally accepted that binding energies below −4.25, −5.0, or −7.0 kcal/mol indicate certain, good, or strong binding activity between the ligands and receptors. The binding energy reflects the likelihood of receptor–ligand interaction. Lower binding energies indicate higher affinity and greater stability between the receptor and ligand. The docking results of binding energies were shown in Table 2, where six pairs exhibited binding energies less than or equal to −7.0 kcal/mol, four pairs had energies less than or equal to −5.0 kcal/mol, and one pair had energy less than or equal to −4.25 kcal/mol. This indicated that most receptor–ligand pairs could bind well and exert their effects. The Top 3 active compounds with the lowest binding energies in the docking process were all rubrosterone, and their binding energies were all less than −7.0 kcal/mol, indicating that rubrosterone could form stable conformations with the target receptors and exert its activity.

### 3.8. Molecular Dynamics Results

#### 3.8.1. Root Mean Square Deviation (RMSD)

**Figure 5:**
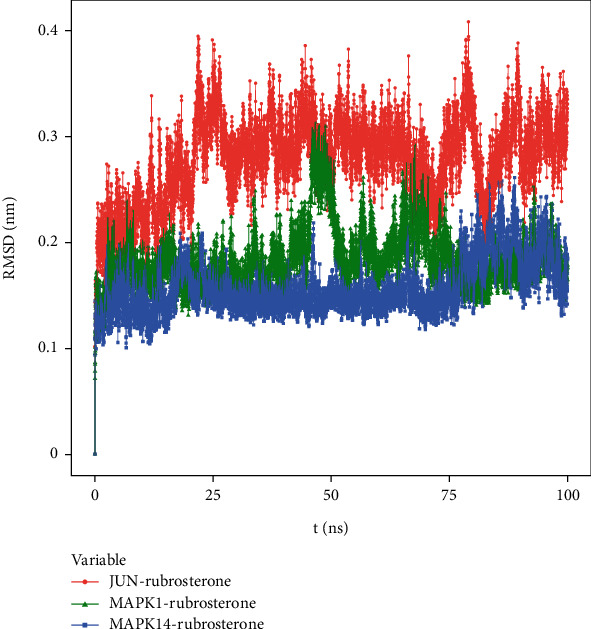
RMSD of receptor–ligand complexes during 100 ns simulation.

RMSD curves in molecular dynamics simulations represented the fluctuations in protein conformation, reflecting the movement of the receptor–ligand complexes. A higher RMSD value indicated greater instability and pronounced fluctuations, while a lower value indicated a more stable complex.  Figure 5 shows that due to the interactions between the complexes and the solvent, the RMSD curves initially exhibited fluctuations. However, RMSD curves of JUN-rubrosterone, MAPK1-rubrosterone, and MAPK14-rubrosterone stabilized after a transient rise, indicating that the protein conformation did not undergo meaningful changes after binding with the small molecule ligands. These suggested relative stability between the two. Comparing RMSD values, JUN-rubrosterone has the highest RMSD, followed by MAPK1-rubrosterone, and MAPK14-rubrosterone has the lowest RMSD. This indicated that the stability of these complexes follows the order of MAPK14-rubrosterone > MAPK1-rubrosterone > JUN-rubrosterone. The results suggested that all proteins could bind and maintain a relatively stable conformation with rubrosterone. The computational results are presented in Figure 5.

#### 3.8.2. Binding Free Energy

Based on the molecular dynamics simulation trajectories, the binding free energy was calculated using the MM-PBSA method, which accurately reflects the binding mode between the target protein and the small molecule (receptor–ligand). Negative values indicated an affinity between the small molecule and the target protein, and lower values indicate stronger binding. The calculated results demonstrated that these small molecules had certain binding energies with their respective proteins, and the affinities were powerful. Among them, the binding affinity of MAPK14-rubrosterone was the highest, with a value of (−60.379 ± 9.714 kJ/mol) (Table 3).

#### 3.8.3. Hydrogen Bond Analysis

**Figure 6:**
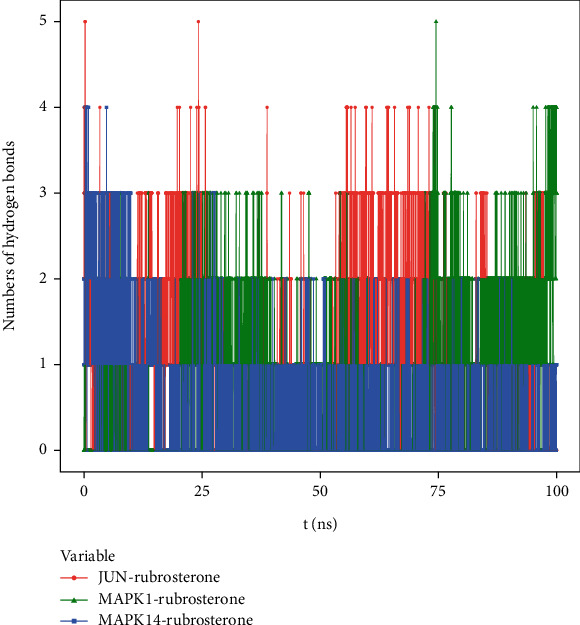
Number of hydrogen bonds in receptor–ligand complexes during 100 ns simulation.

Hydrogen bonding is one of the strongest noncovalent binding interactions. The greater the number of hydrogen bonds, the better the binding effect. The results indicated that three receptor–ligand complexes had similar hydrogen bonds and good binding interactions. The hydrogen bond quantities shown in Figure 6 suggested that the interactions between JUN-rubrosterone and MAPK1-rubrosterone might be primarily mediated by hydrogen bonds. The interaction of MAPK14-rubrosterone, on the other hand, likely occurred through hydrophobic interactions rather than hydrogen bonding.

#### 3.8.4. Stability of Target Proteins at the Residue Level

**Figure 7:**
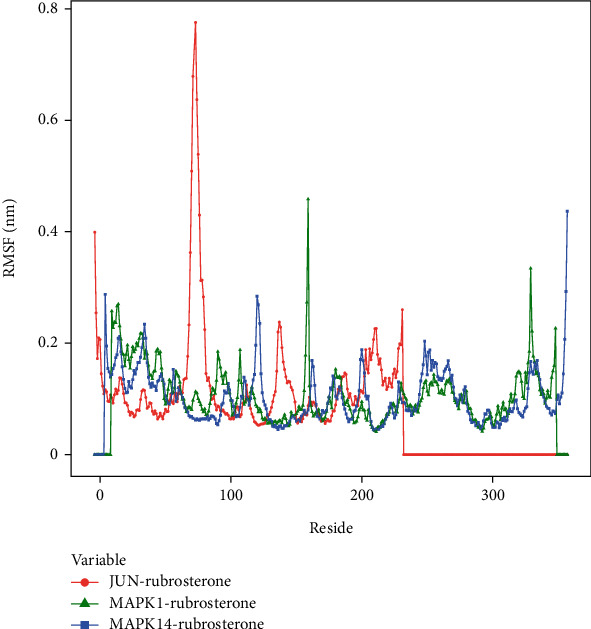
RMSF of receptor–ligand complexes during 100 ns simulation.

To explore the local fluctuations of large molecular proteins at the residue level, the root mean square fluctuation (RMSF) is introduced to reflect the flexibility of proteins during molecular dynamics simulations. Generally, upon binding of drugs to proteins, the flexibility of the proteins decreases, leading to the stabilization of protein structures and facilitating their enzymatic activities. The results indicated that the peak and valley positions of RMSF differ among these complexes, suggesting different effective binding sites for the drug. Therefore, three complexes might exert their effects through distinct mechanisms, which merited further in-depth investigation. The RMSF calculation results are shown in Figure 7.

#### 3.8.5. Radius of Gyration (R(g))

**Figure 8:**
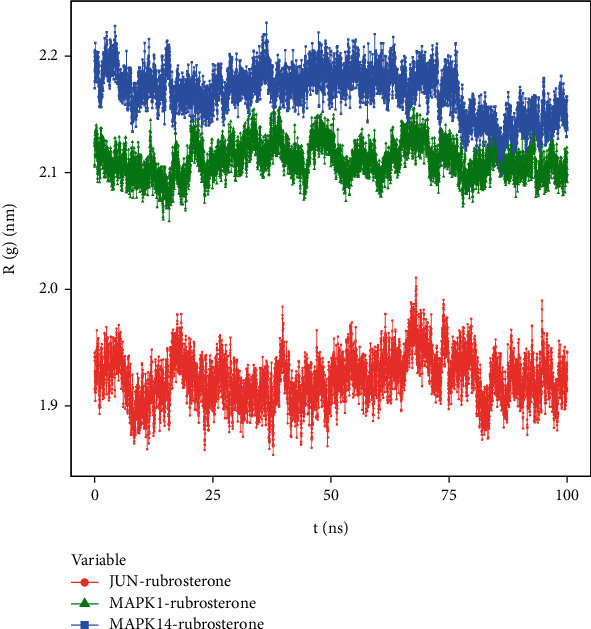
R(g) of receptor–ligand complexes during 100 ns simulation.

R(g) is a curve used to describe the compactness variations in the overall structure of receptor–ligand complexes, and it also reflects the overall constraint of protein structures. The average R(g) values of three receptor–ligand complexes were around two, indicating a high density and tight binding of the system. The results in Figure 8 demonstrated that JUN-rubrosterone, MAPK1-rubrosterone, and MAPK14-rubrosterone all exhibited highly stable R(g), which aligned with the information reflected by the RMSD curve and confirmed the stability of protein conformations.

#### 3.8.6. Solvent-Accessible Surface Area (SASA)

**Figure 9:**
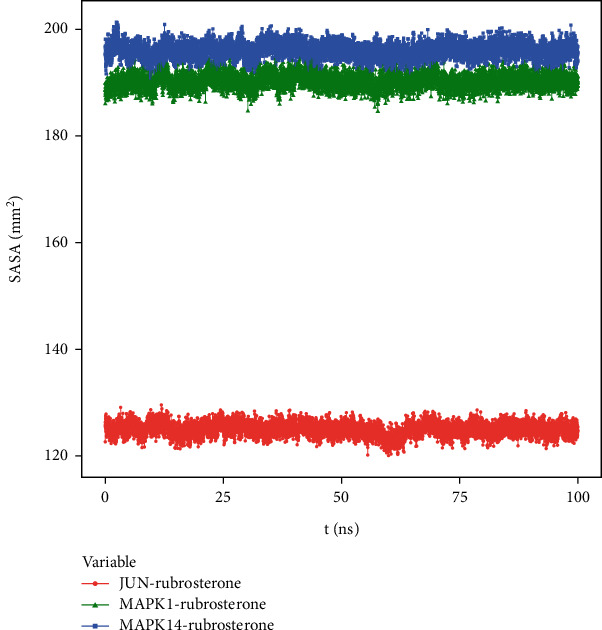
SASA of receptor–ligand complexes during 100 ns simulation.

SASA is calculated based on the interface surrounded by the solvent. Solvents exhibit different behaviors under different conditions, making SASA a useful parameter for studying protein conformational dynamics in solvent environments. The solvent-accessible surface areas of MAPK1-rubrosterone and MAPK14-rubrosterone complexes were similar, suggesting that the impact of solvents on the action of small molecule substances on protein targets was relatively minor. The results are shown in Figure 9.

### 3.9. Effects of High Glucose and CR on Cell Viability

**Figure 10:**
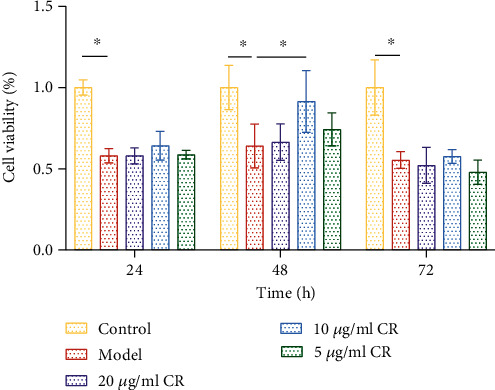
Effect of CR on cell viability. ⁣^∗^*p* < 0.05.

Cell viability was detected using the CCK-8 method to assess the effect of CR on cell viability. As shown in Figure 10, after 24 h of high glucose intervention, the cell viability of the model group decreased by 48.57% compared to the control group (*p* < 0.05). After 48 h of high glucose intervention, the cell viability of the model group decreased by 42.38% compared to the control group (*p* < 0.05). After 72 h of high glucose intervention, the cell viability of the model group decreased by 48.97% compared to the control group (*p* < 0.05). There was no notable difference in the cell viability among three groups. It indicated that high glucose inhibited INS-1 cell viability, and the difference was not significant within the 24–72 h period. Compared to the model group, CR improved cell viability (89.29%) after 48 h of intervention and a drug concentration of 10 *μ*g/mL (*p* < 0.05). These results indicate that CR, at a concentration of 10 *μ*g/mL and an intervention time of 48 h, was the optimal concentration and intervention time to enhance pancreatic *β*-cell viability during high glucose injury.

### 3.10. Insulin Concentration Results

According to the results presented in Table 4, the model group exhibited a vital decrease in insulin levels compared to the control group (*p* < 0.05). This finding suggests that high glucose levels contribute to the reduction in insulin secretion. Conversely, the CR group showed significantly higher insulin levels than the control group (*p* < 0.05), indicating that this intervention enhances insulin secretion, even in high glucose inhibition.

### 3.11. Changes in Mitochondrial Morphology and Apoptosis Levels Among Different Groups

**Figure 11:**
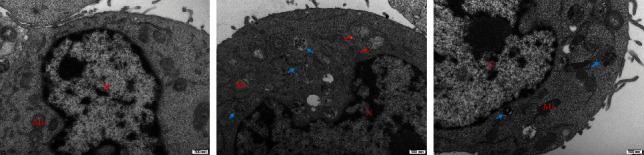
Transmission electron microscopy results of each group. “N” represents the nucleus, and “Mi” represents mitochondria. The blue arrow represents autophagy, and the red arrow represents mitochondrial swelling.

In this study, transmission electron microscopy revealed distinct differences in mitochondrial status and apoptosis levels among three groups (Figure 11). In the control group, mitochondrial structures were intact and distinctive. Many mitochondria exhibited a spherical or elliptical shape with numerous internal cristae and no apparent signs of apoptosis. In the model group, most mitochondria showed some swelling, partial cristae fragmentation and dissolution, uneven electron density in the matrix, an abundance of autophagic structures in the cytoplasm, and a few cells prone to apoptosis, resulting in significantly enlarged mitochondria. In the CR group, the majority of cells exhibited a more continuous and intact mitochondrial outer membrane compared to the model group. The cristae were more organized, and the number of swollen mitochondria decreased, with no apparent tendency towards apoptosis.

### 3.12. CR Affected Relative mRNA Levels of JUN, MAPK1, and MAPK14 in INS-1 Cells Treated With High Glucose

**Figure 12:**
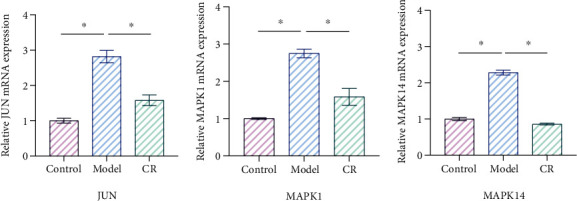
qPCR analysis of JUN, MAPK1, and MAPK14. ⁣^∗^*p* < 0.05.

Bioinformatics analysis showed that JUN, MAPK1, and MAPK14 were core targets of CR in treating T2DM. Furthermore, molecular docking results indicated that the primary active compounds of CR effectively bind to JUN, MAPK1, and MAPK14. Therefore, we selected JUN, MAPK1, and MAPK14 for further qPCR validation to determine whether CR regulates the mRNA expression of JUN, MAPK1, and MAPK14 in INS-1 cells. The qPCR results showed that the mRNA levels of JUN, MAPK1, and MAPK14 were increased in the model group compared to the control group (*p* < 0.05). Compared to the model group, CR (10 *μ*g/mL) contributed to the downregulation of JUN, MAPK1, and MAPK14 expression levels (*p* < 0.05) (Figure 12). Therefore, these results indicate that CR inhibited the expression of JUN, MAPK1, and MAPK14 in a high glucose-induced cell damage model.

## 4. Discussion

This study employed systematic bioinformatics analysis and experimental validation to predict and elucidate the potential molecular mechanisms of CR against T2DM. Based on the screening results, it was found that the therapeutic effects of CR on T2DM were associated with four active compounds, including beta vulgarin, quercetin, beta-sitosterol, and rubrosterone. These findings indicate that the pharmacological effects of CR in T2DM result from these. Quercetin could inhibit pancreatic ferroptosis and pancreatic *β*-cell death, exerting a potential hypoglycemic effect [20]. The therapeutic effect of quercetin on T2DM has been widely studied and recognized. It is worth noting that there is still much research space for the other three active compounds. Beta vulgarin is an emerging antidiabetic drug in recent years. It exerted its blood glucose-controlling effects in a T2DM rat model by reducing blood glucose, increasing insulin sensitivity, and exerting antioxidant effects [21]. Previous in vivo experiments had demonstrated that beta vulgarin could alleviate anxiety and depression in diabetic rats by intervening in dopamine and serotonin receptor–dependent pathways [22]. Beta-sitosterol has been recognized as a herbal nutraceutical with great potential for diabetic management [23]. It inhibited obesity-induced IR by ameliorating inflammatory events in adipose tissue through the downregulation of the nuclear factor kappa-B kinase subunit beta (IKK*β*)/nuclear factor kappa B (NF-*κ*B) and c-Jun-N-terminal kinase (JNK) signaling pathway [24]. Additionally, beta-sitosterol regulated insulin secretion and glucose transporter 4 (GLUT4) expression by activating G-protein-coupled receptor 40 (GPR40) and peroxisome proliferator-activated receptor–gamma (PPAR*γ*) receptors, ultimately reducing blood glucose [25]. Rubrosterone also exhibits antidiabetic bioactivity [26]. Both molecular docking and molecular dynamics results in this study indicate that rubrosterone binds well with JUN, MAPK1, and MAPK14. This suggests that rubrosterone holds great potential as a compound for antidiabetic treatment, representing an area that urgently requires development. However, the specific mechanisms by which rubrosterone exerts its therapeutic effects on T2DM are currently unclear and require further experimental research and clinical evidence. Undoubtedly, our study filled a gap in this field.

PPI analysis revealed that JUN, MAPK1, and MAPK14 were critical targets for treating T2DM with CR. These target genes were crucial proteins in the MAPK signaling pathway family. JUN was an important protein in all cells involved in appropriate responses to stress [27]. Reducing the expression of JUN could reverse T2DM by reducing the expression of inflammatory factors [28], inhibiting pancreatic *β*-cell apoptosis and IR [29, 30]. MAPK1 was closely associated with changes in glucose and normal glucose homeostasis [31, 32]. It has been shown that downregulating the expression of MAPK1 could enzymatically inhibit the activation of proteins related to IR, such as STAT3, and had been proposed as a valuable candidate target for diabetes treatment. Reducing the expression of MAPK14 played a role in glucose and lipid metabolism [33]. Studies have shown that two variations (rs3761980 and rs80028505) associated with diabetic neuropathy were located near MAPK14 [34], and it was associated with an increased risk of diabetic foot ulcer in the GoDARTS (Genetics of Diabetes Audit and Research Tayside, Scotland) project [35]. MAPK1 and MAPK14 have been identified as key proteins regulating mitochondrial autophagy and structure [36]. Overwhelming evidence indicated that T2DM led to dysregulation of autophagy, morphological changes in mitochondria, and cell apoptosis in pancreatic *β*-cells [37, 38]. We have further confirmed this conclusion through transmission electron microscopy, and it was evident that CR improves such stress-induced alterations. In summary, the above discussion suggested that the core targets of CR were of crucial importance in the treatment of diabetes.

We provided the first evidence demonstrating the efficacy of CR in promoting insulin secretion and restoring pancreatic islet function in INS-1 cells impaired by high glucose damage. Furthermore, TEM observations revealed that CR could mitigate cellular apoptosis, restore mitochondrial structure, and modulate autophagy. It is widely acknowledged that cell apoptosis, mitochondrial structure, and cellular autophagy are current research focal points in the context of T2DM treatment. This reaffirmed the tremendous potential of CR in the therapeutic management of T2DM. From the perspective of molecular mechanisms, the core targets (JUN, MAPK1, and MAPK14) were validated through molecular docking, molecular dynamics, and experimental studies. The molecular docking and molecular dynamics results were successful based on the interactions between molecules. Experimental studies confirmed that CR significantly reduced the expression of JUN, MAPK1, and MAPK14, indicating its beneficial effects in protecting pancreatic *β*-cells from high glucose damage. Therefore, we propose that CR exerts a therapeutic effect on T2DM by modulating these targets.

According to the findings from the GO functional enrichment analysis, it was indicated that CR might potentially ameliorate T2DM by modulating various BP, CC, and MF. This regulatory effect on T2DM was likely attributed to the intervention of multiple stress reactions. Furthermore, the identified targets were predominantly situated within the membrane raft and other components, as well as DNA-related transcription factors. In KEGG enrichment analysis, we identified several signaling pathways related to the disease, such as the AGE-RAGE signaling pathway in diabetic complications and the lipid and atherosclerosis signaling pathway, which were closely associated with the occurrence and development of T2DM. Targeting AGEs/RAGE with its ligands-mediated oxidative stress and chronic inflammation was an additional intervention strategy for diabetic kidney disease (DKD). The most substantial evidence supports that hyperglycemia-related AGE formation plays a central role in DKD. Pathologically, the accumulation of AGEs activates RAGE, leading to oxidative stress and inflammation, ultimately resulting in DKD [39]. It should be noted that there is no research available on the treatment of DKD with CR. Atherosclerosis is a vascular disease caused by hyperlipidemia. T2DM promotes atherosclerosis, which can lead to cardiovascular diseases and is a major contributor to the incidence and mortality rates of diabetes-related complications [40]. Therefore, elucidating the underlying mechanisms of T2DM-induced atherosclerosis is prominent for treating and preventing T2DM–related cardiovascular diseases [41]. JUN is involved in the lipid and atherosclerosis signaling pathways [42]. It is reasonable to speculate that CR may play a role in reducing lipid accumulation and mitigating the risk of T2DM-related atherosclerosis.

It is crucial to acknowledge the inherent complexities and potential limitations associated with these computational findings in this study. The current state of bioinformatics technology requires further improvement, and the accuracy and timeliness of database information need to be scientifically validated. The bioinformatics tools used for enrichment analyses rely on databases and algorithms that may have inherent biases or incomplete datasets, which can affect the comprehensiveness and accuracy of pathway predictions. Molecular dynamics simulations are highly dependent on the parameters and force fields chosen for the simulations. Variability in these factors can influence the outcomes and interpretations derived from such simulations. Additionally, active compounds that were not recorded or validated in the database were not included in the analysis of this study. While the findings suggest promising avenues for further research into CR's therapeutic potential in T2DM, comprehensive experimental validations are essential to corroborate these predictions and to better understand the true mechanistic underpinnings. In summary, this study held considerable potential for future development and research significance.

## 5. Conclusion

In conclusion, the antidiabetic properties of CR could be attributed to four active compounds corresponding to 141 hypoglycemic targets. CR promoted insulin secretion, preserved mitochondrial structure integrity, regulated autophagy, and reduced apoptosis in INS-1 cells. JUN, MAPK1, and MAPK14 might be potential therapeutic targets for CR in treating T2DM. Our research findings provide scientific evidence for supporting the clinical application of CR in treating T2DM.

## Figures and Tables

**Table 1:** qPCR primer sequences.

**Gene**	**Forward primer (5**⁣′** to 3**⁣′**)**	**Reverse primer (5**⁣′** to 3**⁣′**)**
JUN	GAACCGCATCGCTGCCT	CATGAGTTGGCACCCACTGTTA
MAPK1	AACCTCCTGCTGAACACCACT	CGTGGCTACATACTCTGTCAAGAAC
MAPK14	CCCGAGCGATACCAGAACCT	TGGCGTGAATGATGGACTGA
GAPDH	CTGGAGAAACCTGCCAAGTATG	GGTGGAAGAATGGGAGTTGCT

**Table 2:** Binding energy for JUN, MAPK1, and MAPK14 with active compounds of CR.

**Hub targets**	**Rubrosterone (kcal/mol)**	**Beta vulgarin (kcal/mol)**	**Quercetin (kcal/mol)**	**Beta-sitosterol (kcal/mol)**
JUN	−8.5	−7.1	−6.4	−5.1
MAPK1	−7.9	−6.2	−4.8	−3.9
MAPK14	−9.7	−7.3	−7.2	−6.7

**Table 3:** Binding free energy and energy value of each receptor–ligand complex.

**Receptor–ligand**	**Van der Waals energy/(kJ·Mol** ^**−1**^ **)**	**Electrostatic energy/(kJ·Mol** ^**−1**^ **)**	**Polar solvation energy/(kJ·Mol** ^**−1**^ **)**	**Nonpolar solvation energy/(kJ·Mol** ^**−1**^ **)**	**Total binding free energy/(kJ·Mol** ^**−1**^ **)**
JUN-rubrosterone	−52.156 ± 28.486	−24.471 ± 25.005	61.061 ± 40.899	−9.695 ± 4.307	−25.261 ± 22.456
MAPK1-rubrosterone	−91.744 ± 13.203	−41.279 ± 15.510	106.574 ± 20.770	−16.057 ± 2.333	−42.506 ± 11.116
MAPK14-rubrosterone	−90.889 ± 6.540	−1.514 ± 6.142	45.197 3 11.151	−13.172 ± 0.568	−60.379 ± 9.714

**Table 4:** Insulin secretion in different groups.

**Group**	**Number of duplicate holes**	**Insulin levels of glucose (mIU/L)**
Control	3	6.164 ± 0.185
Model	3	4.548 ± 0.197^△^
CR	3	5.401 ± 0.179^▲^

^▲^ *p* < 0.05, compared with the model group.

^△^ *p* < 0.05, compared with the control group.

## Data Availability

The original contributions presented in the study were included in the article, and further inquiries could be directed to the corresponding author.

## Supporting Information

Supporting Information 1 Figure S1: the PPI network.

Supporting Information 2 Table S1: the details of beta vulgarin, quercetin, beta-sitosterol, and rubrosterone.

Supporting Information 3 Table S2: 141 common targets of target genes of CR crossed with those of T2DM.
