# Supplementary figures and images for: Bioinformatics Analysis and Experimental Findings Reveal the Therapeutic Actions and Targets of Cyathulae Radix Against Type 2 Diabetes Mellitus
Source: J Diabetes Res. 2024 Nov 5;2024:5521114. doi: 10.1155/2024/5521114 (PMC11557179; doi:10.1155/2024/5521114)

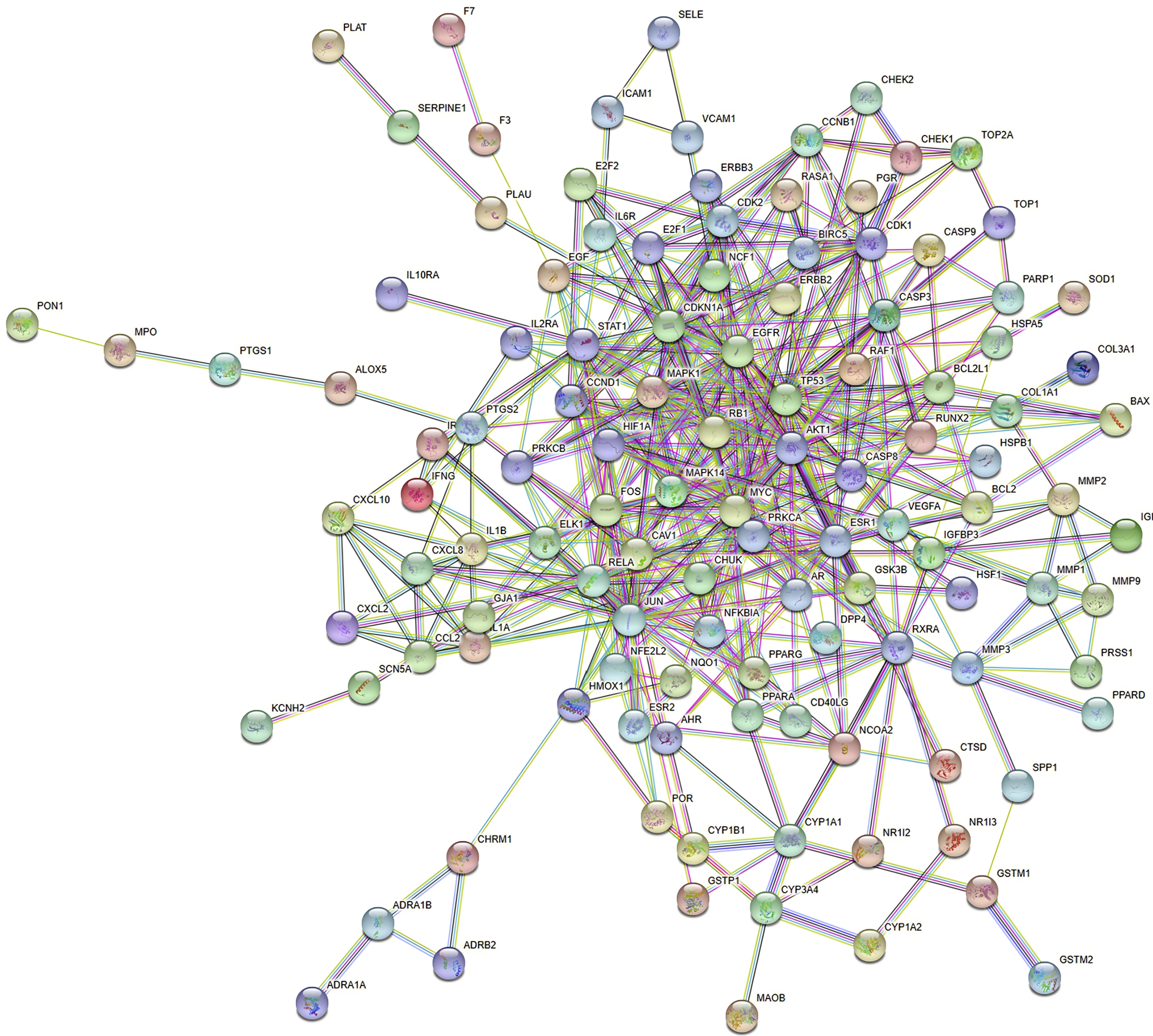

Supplement: Supporting Information 1 — Figure S1: the PPI network. [file 5521114.f1.PDF]
