# Supplementary material for: Bioinformatics Analysis and Experimental Findings Reveal the Therapeutic Actions and Targets of Cyathulae Radix Against Type 2 Diabetes Mellitus
Source: J Diabetes Res. 2024 Nov 5;2024:5521114. doi: 10.1155/2024/5521114 (PMC11557179; doi:10.1155/2024/5521114)
Supplement: Supporting Information 2 — Table S1: the details of beta vulgarin, quercetin, beta-sitosterol, and rubrosterone. [file 5521114.f2.pdf]

	MOL_ID	ob	dl	molecule_name	target_name	Drug
1	MOL012286	68.74957	0.39376	Betavulgarin	Prostaglandin G/H synthase 1	川牛膝筛选
2	MOL012286	68.74957	0.39376	Betavulgarin	Androgen receptor	川牛膝筛选
3	MOL012286	68.74957	0.39376	Betavulgarin	Potassium voltage-gated channel subfamily H member 2	川牛膝筛选
4	MOL012286	68.74957	0.39376	Betavulgarin	Calmodulin	川牛膝筛选
5	MOL012286	68.74957	0.39376	Betavulgarin	Thrombin	川牛膝筛选
6	MOL012286	68.74957	0.39376	Betavulgarin	Coagulation factor VII	川牛膝筛选
7	MOL012286	68.74957	0.39376	Betavulgarin	Estrogen receptor	川牛膝筛选
8	MOL012286	68.74957	0.39376	Betavulgarin	Nitric oxide synthase, inducible	川牛膝筛选
9	MOL012286	68.74957	0.39376	Betavulgarin	Dipeptidyl peptidase IV	川牛膝筛选
10	MOL012286	68.74957	0.39376	Betavulgarin	Mitogen-activated protein kinase 14	川牛膝筛选
11	MOL012286	68.74957	0.39376	Betavulgarin	Glycogen synthase kinase-3 beta	川牛膝筛选
12	MOL012286	68.74957	0.39376	Betavulgarin	Heat shock protein HSP 90	川牛膝筛选
13	MOL012286	68.74957	0.39376	Betavulgarin	Cell division protein kinase 2	川牛膝筛选
14	MOL012286	68.74957	0.39376	Betavulgarin	Sodium channel protein type 5 subunit alpha	川牛膝筛选
15	MOL012286	68.74957	0.39376	Betavulgarin	Trypsin-1	川牛膝筛选
16	MOL012286	68.74957	0.39376	Betavulgarin	Calcium-activated potassium channel subunit alpha 1	川牛膝筛选
17	MOL012286	68.74957	0.39376	Betavulgarin	Peroxisome proliferator activated receptor gamma	川牛膝筛选
18	MOL012286	68.74957	0.39376	Betavulgarin	Prostaglandin G/H synthase 2	川牛膝筛选
19	MOL012286	68.74957	0.39376	Betavulgarin	Carbonic anhydrase II	川牛膝筛选
20	MOL012286	68.74957	0.39376	Betavulgarin	Serine/threonine-protein kinase Chk1	川牛膝筛选
21	MOL012286	68.74957	0.39376	Betavulgarin	Retinoic acid receptor RXR-alpha	川牛膝筛选
22	MOL012286	68.74957	0.39376	Betavulgarin	Estrogen receptor beta	川牛膝筛选
23	MOL012286	68.74957	0.39376	Betavulgarin	Phosphatidylinositol-4,5-bisphosphate 3-kinase catalytic subunit, gamma isoform	川牛膝筛选
24	MOL012286	68.74957	0.39376	Betavulgarin	Ig gamma-1 chain C region	川牛膝筛选
25	MOL012298	32.69189	0.46605	Rubrosterone	Mineralocorticoid receptor	川牛膝筛选
26	MOL000358	36.91391	0.75123	beta-sitosterol	Microtubule-associated protein 2	川牛膝筛选
27	MOL000358	36.91391	0.75123	beta-sitosterol	Protein kinase C alpha type	川牛膝筛选
28	MOL000358	36.91391	0.75123	beta-sitosterol	Transforming growth factor beta-1	川牛膝筛选
29	MOL000358	36.91391	0.75123	beta-sitosterol	Serum paraoxonase/arylesterase 1	川牛膝筛选
30	MOL000358	36.91391	0.75123	beta-sitosterol	Apoptosis regulator Bcl-2	川牛膝筛选
31	MOL000358	36.91391	0.75123	beta-sitosterol	Apoptosis regulator BAX	川牛膝筛选
32	MOL000358	36.91391	0.75123	beta-sitosterol	Caspase-9	川牛膝筛选
33	MOL000358	36.91391	0.75123	beta-sitosterol	Transcription factor AP-1	川牛膝筛选
34	MOL000358	36.91391	0.75123	beta-sitosterol	Caspase-3	川牛膝筛选
35	MOL000358	36.91391	0.75123	beta-sitosterol	Caspase-8	川牛膝筛选
36	MOL000358	36.91391	0.75123	beta-sitosterol	Potassium voltage-gated channel subfamily H member 2	川牛膝筛选
37	MOL000358	36.91391	0.75123	beta-sitosterol	mRNA of PKA Catalytic Subunit C-alpha	川牛膝筛选
38	MOL000358	36.91391	0.75123	beta-sitosterol	Dopamine D1 receptor	川牛膝筛选
39	MOL000358	36.91391	0.75123	beta-sitosterol	Muscarinic acetylcholine receptor M3	川牛膝筛选
40	MOL000358	36.91391	0.75123	beta-sitosterol	Muscarinic acetylcholine receptor M1	川牛膝筛选
41	MOL000358	36.91391	0.75123	beta-sitosterol	Sodium channel protein type 5 subunit alpha	川牛膝筛选
42	MOL000358	36.91391	0.75123	beta-sitosterol	Gamma-aminobutyric-acid receptor alpha-2 subunit	川牛膝筛选
43	MOL000358	36.91391	0.75123	beta-sitosterol	Progesterone receptor	川牛膝筛选
44	MOL000358	36.91391	0.75123	beta-sitosterol	Nuclear receptor coactivator 2	川牛膝筛选
45	MOL000358	36.91391	0.75123	beta-sitosterol	Prostaglandin G/H synthase 1	川牛膝筛选
46	MOL000358	36.91391	0.75123	beta-sitosterol	Prostaglandin G/H synthase 2	川牛膝筛选
47	MOL000358	36.91391	0.75123	beta-sitosterol	Heat shock protein HSP 90	川牛膝筛选
48	MOL000358	36.91391	0.75123	beta-sitosterol	Phosphatidylinositol-4,5-bisphosphate 3-kinase catalytic subunit, gamma isoform	川牛膝筛选
49	MOL000358	36.91391	0.75123	beta-sitosterol	Alpha-1B adrenergic receptor	川牛膝筛选
50	MOL000358	36.91391	0.75123	beta-sitosterol	Beta-2 adrenergic receptor	川牛膝筛选
51	MOL000358	36.91391	0.75123	beta-sitosterol	Neuronal acetylcholine receptor subunit alpha-2	川牛膝筛选
52	MOL000358	36.91391	0.75123	beta-sitosterol	Sodium-dependent serotonin transporter	川牛膝筛选
53	MOL000358	36.91391	0.75123	beta-sitosterol	Mu-type opioid receptor	川牛膝筛选
54	MOL000358	36.91391	0.75123	beta-sitosterol	Gamma-aminobutyric acid receptor subunit alpha-1	川牛膝筛选
55	MOL000358	36.91391	0.75123	beta-sitosterol	Neuronal acetylcholine receptor protein, alpha-7 chain	川牛膝筛选
56	MOL000358	36.91391	0.75123	beta-sitosterol	Cytochrome P450-cam	川牛膝筛选
57	MOL000358	36.91391	0.75123	beta-sitosterol	Muscarinic acetylcholine receptor M4	川牛膝筛选
58	MOL000358	36.91391	0.75123	beta-sitosterol	CGMP-inhibited 3',5'-cyclic phosphodiesterase A	川牛膝筛选
59	MOL000358	36.91391	0.75123	beta-sitosterol	5-hydroxytryptamine 2A receptor	川牛膝筛选
60	MOL000358	36.91391	0.75123	beta-sitosterol	Gamma-aminobutyric-acid receptor alpha-5 subunit	川牛膝筛选
61	MOL000358	36.91391	0.75123	beta-sitosterol	Alpha-1A adrenergic receptor	川牛膝筛选
62	MOL000358	36.91391	0.75123	beta-sitosterol	Gamma-aminobutyric-acid receptor alpha-3 subunit	川牛膝筛选
63	MOL000358	36.91391	0.75123	beta-sitosterol	Muscarinic acetylcholine receptor M2	川牛膝筛选
64	MOL000098	46.43335	0.27525	quercetin	Coagulation factor Xa	川牛膝筛选
65	MOL000098	46.43335	0.27525	quercetin	Beta-2 adrenergic receptor	川牛膝筛选
66	MOL000098	46.43335	0.27525	quercetin	Stromelysin-1	川牛膝筛选
67	MOL000098	46.43335	0.27525	quercetin	mRNA of PKA Catalytic Subunit C-alpha	川牛膝筛选
68	MOL000098	46.43335	0.27525	quercetin	Coagulation factor VII	川牛膝筛选
69	MOL000098	46.43335	0.27525	quercetin	Nitric-oxide synthase, endothelial	川牛膝筛选
70	MOL000098	46.43335	0.27525	quercetin	Dipeptidyl peptidase IV	川牛膝筛选
71	MOL000098	46.43335	0.27525	quercetin	Aldose reductase	川牛膝筛选
72	MOL000098	46.43335	0.27525	quercetin	Trypsin-1	川牛膝筛选
73	MOL000098	46.43335	0.27525	quercetin	DNA topoisomerase II	川牛膝筛选
74	MOL000098	46.43335	0.27525	quercetin	Thrombin	川牛膝筛选
75	MOL000098	46.43335	0.27525	quercetin	Potassium voltage-gated channel subfamily H member 2	川牛膝筛选
76	MOL000098	46.43335	0.27525	quercetin	Sodium channel protein type 5 subunit alpha	川牛膝筛选
77	MOL000098	46.43335	0.27525	quercetin	Vascular endothelial growth factor A	川牛膝筛选
78	MOL000098	46.43335	0.27525	quercetin	G1/S-specific cyclin-D1	川牛膝筛选
79	MOL000098	46.43335	0.27525	quercetin	Apoptosis regulator Bcl-2	川牛膝筛选
80	MOL000098	46.43335	0.27525	quercetin	Bcl-2-like protein 1	川牛膝筛选
81	MOL000098	46.43335	0.27525	quercetin	Proto-oncogene c-Fos	川牛膝筛选
82	MOL000098	46.43335	0.27525	quercetin	Cyclin-dependent kinase inhibitor 1	川牛膝筛选
83	MOL000098	46.43335	0.27525	quercetin	Retinoic acid receptor RXR-alpha	川牛膝筛选
84	MOL000098	46.43335	0.27525	quercetin	Acetylcholinesterase	川牛膝筛选
85	MOL000098	46.43335	0.27525	quercetin	Gamma-aminobutyric acid receptor subunit alpha-1	川牛膝筛选
86	MOL000098	46.43335	0.27525	quercetin	Amine oxidase [flavin-containing] B	川牛膝筛选
87	MOL000098	46.43335	0.27525	quercetin	Transcription factor p65	川牛膝筛选
88	MOL000098	46.43335	0.27525	quercetin	Epidermal growth factor receptor	川牛膝筛选
89	MOL000098	46.43335	0.27525	quercetin	RAC-alpha serine/threonine-protein kinase	川牛膝筛选
90	MOL000098	46.43335	0.27525	quercetin	Prostaglandin G/H synthase 1	川牛膝筛选
91	MOL000098	46.43335	0.27525	quercetin	Androgen receptor	川牛膝筛选
92	MOL000098	46.43335	0.27525	quercetin	Peroxisome proliferator activated receptor gamma	川牛膝筛选
93	MOL000098	46.43335	0.27525	quercetin	Prostaglandin G/H synthase 2	川牛膝筛选
94	MOL000098	46.43335	0.27525	quercetin	Heat shock protein HSP 90	川牛膝筛选
95	MOL000098	46.43335	0.27525	quercetin	Phosphatidylinositol-4,5-bisphosphate 3-kinase catalytic subunit, gamma isoform	川牛膝筛选
96	MOL000098	46.43335	0.27525	quercetin	Nuclear receptor coactivator 2	川牛膝筛选
97	MOL000098	46.43335	0.27525	quercetin	Activator of 90 kDa heat shock protein ATPase homolog 1	川牛膝筛选
98	MOL000098	46.43335	0.27525	quercetin	Caspase-3	川牛膝筛选
99	MOL000098	46.43335	0.27525	quercetin	Cellular tumor antigen p53	川牛膝筛选
100	MOL000098	46.43335	0.27525	quercetin	ETS domain-containing protein Elk-1	川牛膝筛选
101	MOL000098	46.43335	0.27525	quercetin	NF-kappa-B inhibitor alpha	川牛膝筛选
102	MOL000098	46.43335	0.27525	quercetin	NADPH--cytochrome P450 reductase	川牛膝筛选
103	MOL000098	46.43335	0.27525	quercetin	Ornithine decarboxylase	川牛膝筛选
104	MOL000098	46.43335	0.27525	quercetin	Xanthine dehydrogenase/oxidase	川牛膝筛选
105	MOL000098	46.43335	0.27525	quercetin	Caspase-8	川牛膝筛选
106	MOL000098	46.43335	0.27525	quercetin	DNA topoisomerase 1	川牛膝筛选
107	MOL000098	46.43335	0.27525	quercetin	RAF proto-oncogene serine/threonine-protein kinase	川牛膝筛选
108	MOL000098	46.43335	0.27525	quercetin	Superoxide dismutase [Cu-Zn]	川牛膝筛选
109	MOL000098	46.43335	0.27525	quercetin	Protein kinase C alpha type	川牛膝筛选
110	MOL000098	46.43335	0.27525	quercetin	Interstitial collagenase	川牛膝筛选
111	MOL000098	46.43335	0.27525	quercetin	Hypoxia-inducible factor 1-alpha	川牛膝筛选
112	MOL000098	46.43335	0.27525	quercetin	Signal transducer and activator of transcription 1-alpha/beta	川牛膝筛选
113	MOL000098	46.43335	0.27525	quercetin	Protein CBFA2T1	川牛膝筛选
114	MOL000098	46.43335	0.27525	quercetin	Probable E3 ubiquitin-protein ligase HERC5	川牛膝筛选
115	MOL000098	46.43335	0.27525	quercetin	Cell division control protein 2 homolog	川牛膝筛选
116	MOL000098	46.43335	0.27525	quercetin	78 kDa glucose-regulated protein	川牛膝筛选
117	MOL000098	46.43335	0.27525	quercetin	Receptor tyrosine-protein kinase erbB-2	川牛膝筛选
118	MOL000098	46.43335	0.27525	quercetin	Peroxisome proliferator-activated receptor gamma	川牛膝筛选
119	MOL000098	46.43335	0.27525	quercetin	Acetyl-CoA carboxylase 1	川牛膝筛选
120	MOL000098	46.43335	0.27525	quercetin	Heme oxygenase 1	川牛膝筛选
121	MOL000098	46.43335	0.27525	quercetin	Cytochrome P450 3A4	川牛膝筛选
122	MOL000098	46.43335	0.27525	quercetin	Cytochrome P450 1A2	川牛膝筛选
123	MOL000098	46.43335	0.27525	quercetin	Caveolin-1	川牛膝筛选
124	MOL000098	46.43335	0.27525	quercetin	Myc proto-oncogene protein	川牛膝筛选
125	MOL000098	46.43335	0.27525	quercetin	Tissue factor	川牛膝筛选
126	MOL000098	46.43335	0.27525	quercetin	Gap junction alpha-1 protein	川牛膝筛选
127	MOL000098	46.43335	0.27525	quercetin	Cytochrome P450 1A1	川牛膝筛选
128	MOL000098	46.43335	0.27525	quercetin	Intercellular adhesion molecule 1	川牛膝筛选
129	MOL000098	46.43335	0.27525	quercetin	Interleukin-1 beta	川牛膝筛选
130	MOL000098	46.43335	0.27525	quercetin	C-C motif chemokine 2	川牛膝筛选
131	MOL000098	46.43335	0.27525	quercetin	E-selectin	川牛膝筛选
132	MOL000098	46.43335	0.27525	quercetin	Vascular cell adhesion protein 1	川牛膝筛选
133	MOL000098	46.43335	0.27525	quercetin	Prostaglandin E2 receptor EP3 subtype	川牛膝筛选
134	MOL000098	46.43335	0.27525	quercetin	Interleukin-8	川牛膝筛选
135	MOL000098	46.43335	0.27525	quercetin	Protein kinase C beta type	川牛膝筛选
136	MOL000098	46.43335	0.27525	quercetin	Baculoviral IAP repeat-containing protein 5	川牛膝筛选
137	MOL000098	46.43335	0.27525	quercetin	Dual oxidase 2	川牛膝筛选
138	MOL000098	46.43335	0.27525	quercetin	Nitric oxide synthase, endothelial	川牛膝筛选
139	MOL000098	46.43335	0.27525	quercetin	Heat shock protein beta-1	川牛膝筛选
140	MOL000098	46.43335	0.27525	quercetin	Transforming growth factor beta-1	川牛膝筛选
141	MOL000098	46.43335	0.27525	quercetin	Estrogen sulfotransferase	川牛膝筛选
142	MOL000098	46.43335	0.27525	quercetin	Maltase-glucoamylase, intestinal	川牛膝筛选
143	MOL000098	46.43335	0.27525	quercetin	Interleukin-2	川牛膝筛选
144	MOL000098	46.43335	0.27525	quercetin	Nuclear receptor subfamily 1 group I member 2	川牛膝筛选
145	MOL000098	46.43335	0.27525	quercetin	Cytochrome P450 1B1	川牛膝筛选
146	MOL000098	46.43335	0.27525	quercetin	G2/mitotic-specific cyclin-B1	川牛膝筛选
147	MOL000098	46.43335	0.27525	quercetin	Tissue-type plasminogen activator	川牛膝筛选
148	MOL000098	46.43335	0.27525	quercetin	Thrombomodulin	川牛膝筛选
149	MOL000098	46.43335	0.27525	quercetin	Plasminogen activator inhibitor 1	川牛膝筛选
150	MOL000098	46.43335	0.27525	quercetin	Collagen alpha-1(I) chain	川牛膝筛选
151	MOL000098	46.43335	0.27525	quercetin	Interferon gamma	川牛膝筛选
152	MOL000098	46.43335	0.27525	quercetin	Arachidonate 5-lipoxygenase	川牛膝筛选
153	MOL000098	46.43335	0.27525	quercetin	Phosphatidylinositol-3,4,5-trisphosphate 3-phosphatase and dual-specificity protein	川牛膝筛选
154	MOL000098	46.43335	0.27525	quercetin	Interleukin-1 alpha	川牛膝筛选
155	MOL000098	46.43335	0.27525	quercetin	Myeloperoxidase	川牛膝筛选
156	MOL000098	46.43335	0.27525	quercetin	DNA topoisomerase 2-alpha	川牛膝筛选
157	MOL000098	46.43335	0.27525	quercetin	Neutrophil cytosol factor 1	川牛膝筛选
158	MOL000098	46.43335	0.27525	quercetin	ATP-binding cassette sub-family G member 2	川牛膝筛选
159	MOL000098	46.43335	0.27525	quercetin	Hyaluronan synthase 2	川牛膝筛选
160	MOL000098	46.43335	0.27525	quercetin	Glutathione S-transferase P	川牛膝筛选
161	MOL000098	46.43335	0.27525	quercetin	Nuclear factor erythroid 2-related factor 2	川牛膝筛选
162	MOL000098	46.43335	0.27525	quercetin	NAD(P)H dehydrogenase [quinone] 1	川牛膝筛选
163	MOL000098	46.43335	0.27525	quercetin	Poly [ADP-ribose] polymerase 1	川牛膝筛选
164	MOL000098	46.43335	0.27525	quercetin	Aryl hydrocarbon receptor	川牛膝筛选
165	MOL000098	46.43335	0.27525	quercetin	26S proteasome non-ATPase regulatory subunit 3	川牛膝筛选
166	MOL000098	46.43335	0.27525	quercetin	Solute carrier family 2, facilitated glucose transporter member 4	川牛膝筛选
167	MOL000098	46.43335	0.27525	quercetin	Collagen alpha-1(III) chain	川牛膝筛选
168	MOL000098	46.43335	0.27525	quercetin	DNA gyrase subunit B	川牛膝筛选
169	MOL000098	46.43335	0.27525	quercetin	C-X-C motif chemokine 11	川牛膝筛选
170	MOL000098	46.43335	0.27525	quercetin	C-X-C motif chemokine 2	川牛膝筛选
171	MOL000098	46.43335	0.27525	quercetin	DDB1- and CUL4-associated factor 5	川牛膝筛选
172	MOL000098	46.43335	0.27525	quercetin	Nuclear receptor subfamily 1 group I member 3	川牛膝筛选
173	MOL000098	46.43335	0.27525	quercetin	Serine/threonine-protein kinase Chk2	川牛膝筛选
174	MOL000098	46.43335	0.27525	quercetin	Insulin receptor	川牛膝筛选
175	MOL000098	46.43335	0.27525	quercetin	Claudin-4	川牛膝筛选
176	MOL000098	46.43335	0.27525	quercetin	Peroxisome proliferator-activated receptor alpha	川牛膝筛选
177	MOL000098	46.43335	0.27525	quercetin	Peroxisome proliferator-activated receptor delta	川牛膝筛选
178	MOL000098	46.43335	0.27525	quercetin	Heat shock factor protein 1	川牛膝筛选
179	MOL000098	46.43335	0.27525	quercetin	C-reactive protein	川牛膝筛选
180	MOL000098	46.43335	0.27525	quercetin	C-X-C motif chemokine 10	川牛膝筛选
181	MOL000098	46.43335	0.27525	quercetin	Inhibitor of nuclear factor kappa-B kinase subunit alpha	川牛膝筛选
182	MOL000098	46.43335	0.27525	quercetin	Osteopontin	川牛膝筛选
183	MOL000098	46.43335	0.27525	quercetin	Runt-related transcription factor 2	川牛膝筛选
184	MOL000098	46.43335	0.27525	quercetin	Ras association domain-containing protein 1	川牛膝筛选
185	MOL000098	46.43335	0.27525	quercetin	Transcription factor E2F1	川牛膝筛选
186	MOL000098	46.43335	0.27525	quercetin	Transcription factor E2F2	川牛膝筛选
187	MOL000098	46.43335	0.27525	quercetin	Prostatic acid phosphatase	川牛膝筛选
188	MOL000098	46.43335	0.27525	quercetin	Cathepsin D	川牛膝筛选
189	MOL000098	46.43335	0.27525	quercetin	Insulin-like growth factor-binding protein 3	川牛膝筛选
190	MOL000098	46.43335	0.27525	quercetin	Insulin-like growth factor II	川牛膝筛选
191	MOL000098	46.43335	0.27525	quercetin	CD40 ligand	川牛膝筛选
192	MOL000098	46.43335	0.27525	quercetin	Interferon regulatory factor 1	川牛膝筛选
193	MOL000098	46.43335	0.27525	quercetin	Receptor tyrosine-protein kinase erbB-3	川牛膝筛选
194	MOL000098	46.43335	0.27525	quercetin	Serum paraoxonase/arylesterase 1	川牛膝筛选
195	MOL000098	46.43335	0.27525	quercetin	Type I iodothyronine deiodinase	川牛膝筛选
196	MOL000098	46.43335	0.27525	quercetin	Procollagen C-endopeptidase enhancer 1	川牛膝筛选
197	MOL000098	46.43335	0.27525	quercetin	Puromycin-sensitive aminopeptidase	川牛膝筛选
198	MOL000098	46.43335	0.27525	quercetin	Hexokinase-2	川牛膝筛选
199	MOL000098	46.43335	0.27525	quercetin	Homeobox protein Nkx-3.1	川牛膝筛选
200	MOL000098	46.43335	0.27525	quercetin	Ras GTPase-activating protein 1	川牛膝筛选
201	MOL000098	46.43335	0.27525	quercetin	Peroxidase C1A	川牛膝筛选
202	MOL000098	46.43335	0.27525	quercetin	Glutathione S-transferase Mu 1	川牛膝筛选
203	MOL000098	46.43335	0.27525	quercetin	Glutathione S-transferase Mu 2	川牛膝筛选
204	MOL000098	46.43335	0.27525	quercetin	Eukaryotic translation initiation factor 6	川牛膝筛选
205	MOL000098	46.43335	0.27525	quercetin	Apoptosis regulator BAX	川牛膝筛选
206	MOL000098	46.43335	0.27525	quercetin	Caspase-9	川牛膝筛选
207	MOL000098	46.43335	0.27525	quercetin	Urokinase-type plasminogen activator	川牛膝筛选
208	MOL000098	46.43335	0.27525	quercetin	72 kDa type IV collagenase	川牛膝筛选
209	MOL000098	46.43335	0.27525	quercetin	Matrix metalloproteinase-9	川牛膝筛选
210	MOL000098	46.43335	0.27525	quercetin	Mitogen-activated protein kinase 1	川牛膝筛选
211	MOL000098	46.43335	0.27525	quercetin	Interleukin-10	川牛膝筛选
212	MOL000098	46.43335	0.27525	quercetin	Pro-epidermal growth factor	川牛膝筛选
213	MOL000098	46.43335	0.27525	quercetin	Retinoblastoma-associated protein	川牛膝筛选
214	MOL000098	46.43335	0.27525	quercetin	Tumor necrosis factor	川牛膝筛选
215	MOL000098	46.43335	0.27525	quercetin	Transcription factor AP-1	川牛膝筛选
216	MOL000098	46.43335	0.27525	quercetin	Interleukin-6	川牛膝筛选
217	MOL000098	46.43335	0.27525	quercetin	Cyclin-dependent kinase inhibitor 2A, isoforms 1/2/3	川牛膝筛选
