# Supplementary material for: Bioinformatics Analysis and Experimental Findings Reveal the Therapeutic Actions and Targets of Cyathulae Radix Against Type 2 Diabetes Mellitus
Source: J Diabetes Res. 2024 Nov 5;2024:5521114. doi: 10.1155/2024/5521114 (PMC11557179; doi:10.1155/2024/5521114)
Supplement: Supporting Information 3 — Table S2: 141 common targets of target genes of CR crossed with those of T2DM. [file 5521114.f3.pdf]

PTGS1
AR
KCNH2
CAMKMT
F7
ESR1
DPP4
MAPK14
GSK3B
CDK2
SCN5A
PRSS1
PPARG
PTGS2
CA2
CHEK1
RXRA
ESR2
NR3C2
MAP2
PRKCA
PON1
BCL2
BAX
CASP9
JUN
CASP3
CASP8
DRD1
CHRM3
CHRM1
PGR
NCOA2
ADRA1B
ADRB2
SLC6A4
OPRM1
GABRA1
ADRA1A
CHRM2
MMP3
AKR1B1
VEGFA
CCND1
BCL2L1
FOS
CDKN1A
ACHE
MAOB
RELA
EGFR
AKT1
TP53
ELK1

NFKBIA
POR
ODC1
TOP1
RAF1
SOD1
MMP1
HIF1A
STAT1
RUNX1T1
CDK1
HSPA5
ERBB2
ACACA
HMOX1
CYP3A4
CYP1A2
CAV1
MYC
F3
GJA1
CYP1A1
ICAM1
IL1B
CCL2
SELE
VCAM1
PTGER3
CXCL8
PRKCB
BIRC5
DUOX2
HSPB1
IL2RA
NR1I2
CYP1B1
CCNB1
PLAT
THBD
SERPINE1
COL1A1
IFNG
ALOX5
IL1A
MPO
TOP2A
NCF1
ABCG2
HAS2
GSTP1
NFE2L2
NQO1
PARP1
AHR

COL3A1
CXCL2
NR1I3
CHEK2
PPARA
PPARD
HSF1
CXCL10
CHUK
SPP1
RUNX2
RASSF1
E2F1
E2F2
CTSD
IGFBP3
IGF2
CD40LG
IRF1
ERBB3
DIO1
HK2
RASA1
GSTM1
GSTM2
PLAU
MMP2
MMP9
MAPK1
IL10RA
EGF
RB1
IL6R
